# Comparison of the cumulative exposure to four measures of blood pressure for predicting cardiovascular disease risk in the Chinese Uyghurs

**DOI:** 10.1186/s12889-025-22069-9

**Published:** 2025-03-31

**Authors:** Jing Cheng, Bo Yang, Ru-lin Ma, Jia He, Dong-sheng Rui, Yu Li, Xiang-hui Zhang, Le-yao Jian, Jia-hang Li, Shu-xia Guo, Heng Guo

**Affiliations:** 1 Department of Public Health, Shihezi University School of Medicine, North 2th Road, Shihezi, Xinjiang 832003 China; 2 Key Laboratory for Prevention and Control of Emerging Infectious Diseases and Public Health Security, The Xinjiang Production and Construction Corps, Shihezi University, Shihezi, Xinjiang 832000 China; 3 Medical College, Hexi University, Zhangye, Gansu 734000 China

**Keywords:** Systolic blood pressure, Diastolic blood pressure, Mean arterial pressure, Pulse pressure, Cumulative exposure, Cardiovascular disease, Prediction

## Abstract

**Objective:**

This study aimed to explore and compare the role of cumulative exposure to four blood pressure (BP) markers [systolic blood pressure (SBP), diastolic blood pressure (DBP), mean arterial pressure (MAP), and pulse pressure (PP)] in predicting cardiovascular disease (CVD) risk in the Uyghur population.

**Methods:**

We recruited 3,553 Uyghurs from Tumxuk City, and conducted blood pressure measurements on them at least three times, with a minimum interval of two years between consecutive measurements. Cumulative BP was defined as the sum of the product of the average BP between consecutive examinations and the time interval between visits. Cox proportional hazard models and restricted cubic spline (RCS) analysis were used to estimate the association between cumBP and CVD risk. The incremental predictive value of cumBP was further assessed using the net reclassification index (NRI) and integrated discrimination improvement (IDI).

**Results:**

Over a median follow-up of 6.29 years, 383 (10.78%) incidents of CVD occurred. All four cumBP markers were associated with CVD risk, with cumulative SBP (cumSBP) and cumulative PP (cumPP) showing the strongest associations. For each 1-SD increase in cumSBP and cumPP, the CVD risk increased by 31% [HR (95% CI): 1.310 (1.153, 1.489)] and 28% [HR (95% CI): 1.284 (1.132, 1.457)], respectively. Additionally, 1-SD values corresponded to 107.90 mmHg·years for cumSBP and 65.33 mmHg·years for cumPP. RCS analysis showed a linear relationship between cumBP and CVD risk. CumSBP provided the best incremental predictive value for CVD after adding cumSBP to the conventional model, improving the NRI by 0.126 (*P* = 0.019) and the IDI by 0.009 (*P* = 0.001). Although cumulative MAP and cumulative PP also improved the predictive capabilities to varying degrees, the effect sizes were smaller than those of cumSBP.

**Conclusion:**

All four cumBP markers were significantly associated with CVD risk in this population. Compared with the other three cumBP measures, cumSBP had the strongest association with CVD events and provided a superior incremental predictive value for CVD events.

**Supplementary Information:**

The online version contains supplementary material available at 10.1186/s12889-025-22069-9.

## Introduction

Hypertension is a major modifiable risk factor for cardiovascular disease (CVD), with approximately 54% of strokes and 47% of ischaemic heart disease worldwide attributable to high blood pressure (BP) [1, 2]. BP is a dynamic condition that fluctuates over time, and assessing it based on a single time point is susceptible to various factors such as age, lifestyle, and antihypertensive medications. Thus, the disease risk associated with long-term exposure to elevated BP cannot be adequately reflected by a single BP measurement [3–5]. Instead, cumulative BP (cumBP) provides a comprehensive assessment of the severity and duration of BP exposure. It is significantly associated with CVD risk [5–7] and can also improve the accuracy of CVD risk prediction models [5, 6].

Blood pressure consists of two primary components: a steady component estimated by mean arterial pressure (MAP) and a pulsatile component estimated by pulse pressure (PP), both of which are risk factors for CVD [7, 8]. Notably, MAP has been found to be superior to systolic blood pressure (SBP) and diastolic blood pressure (DBP) in predicting CVD risk [8], and cumulative MAP (cumMAP) exposure is similarly identified as a risk factor for CVD [9, 10]. Data from the Framingham Heart Study indicate that PP provides crucial predictive value for coronary heart disease events in elderly adults [11, 12], whereas studies on cumulative PP (cumPP) exposure are relatively scarce. The majority of current research on the relationship between cumBP and CVD outcomes has focused on cumulative systolic blood pressure (cumSBP) and cumulative diastolic blood pressure (cumDBP) [4–6, 13, 14]. However, it remains unclear which measures of cumBP are more effective in predicting CVD risk.

In this study, the Uyghur population in southern Xinjiang, China, was selected as the study population. Their preference for high intakes of salty and fatty foods has led to a heightened prevalence of obesity and hypertension in this population [15, 16], putting them at higher risk of CVD [17]. However, there has been limited research examining the association between cumBP exposure and CVD risk within this population. Therefore, we aimed to assess and compare the role of four cumBP measurements (including cumSBP, cumDBP, cumMAP, and cumPP) in predicting CVD risk in this population.

## Methods

### Study population

Cluster random sampling was conducted in a predominantly Uyghur regiment in the southern Xinjiang region. Tumxuk City was selected as the study site. A total of 12,813 individuals participated in the baseline survey. The inclusion criteria were: (1) Uyghur permanent residents (residence ≥ 1 year). (2) Age ≥ 18 years. (3) Those with three or more BP measurements with an interval of ≥ 2 years between two adjacent measurements. The exclusion criteria were: (1) Those out of town for a long time, mobile population, those with serious illness, unconscious, unwilling to cooperate, and pregnant women. (2) Those with missing follow-up information. (3) Patients with CVD at baseline. Finally, 3,553 people were included in this study according to the inclusion and exclusion criteria.

This investigation was approved by the Ethics Review Committee of the First Affiliated Hospital of Shihezi University School of Medicine (No. 2016–121-01). All participants signed informed consent before taking part in this study.

### Data collection and definitions

Information regarding the general demographic and clinical characteristics, including age, gender, lifestyle, past disease history, and family history, was collected using a standardized questionnaire. Smoking was defined as smoking more than 100 cigarettes ever [18]. Drinking was defined as drinking alcoholic beverages at least twice a month [19]. Body mass index (BMI) was calculated as weight (kg)/height^2^ (m^2^). The physical examiners used uniform standards for measurements, primarily including height, weight, and blood pressure. Smoking and consumption of tea or coffee were prohibited for 30 min prior to BP measurement. Subjects were instructed to sit and rest for 5 min beforehand. Subsequently, a calibrated Omron upper arm electronic blood pressure monitor (model HEM-7051) was used to measure brachial blood pressure. The measurement was repeated after an interval of 2–3 min, and the average of the two readings was taken as the final blood pressure value.

All participants fasted overnight before blood sample collection. All biochemical parameters, including total cholesterol (TC), triglycerides (TG), high-density lipoprotein cholesterol (HDL-C), low-density lipoprotein cholesterol (LDL-C), and fasting blood glucose (FBG), were detected by automatic biochemical analyzers (OLYMPUS AU 2700, Olympus Diagnostics, Hamburg, Germany) in the Laboratory Department of the First Affiliated Hospital of Shihezi University School of Medicine.

Diabetes was defined as FBG ≥ 7.0 mmol/L, use of glucose-lowering medication, or a self-reported history of diabetes. A family history of CVD was defined as a history of CVD in one of their father, mother, or siblings.

### Calculation of cumulative blood pressure

MAP was calculated as (SBP + 2 × DBP)/3 [20], and PP was calculated as (SBP—DBP). Cumulative BP was defined as the sum of the product of the average BP for each pair of consecutive examinations and the time interval between two successive surveys [4, 10]. CumBP = [(BP_1_ + BP_2_)/2 × time_1–2_ + (BP_2_ + BP_3_)/2 × time_2–3_], where BP_1_, BP_2_, and BP_3_ indicate the BP values at the baseline, second, and third physical examinations, respectively, and time_1–2_ and time_2–3_ indicate the time intervals between consecutive visits in years. The mean of time_1–2_ and time_2–3_ was 2.75 years.

### Observational follow-up and assessment of outcomes

The baseline survey was conducted in September 2016 to collect information about questionnaires, blood biochemical indicators, physical examinations, hospital medical records, and social security information. Four follow-up visits were conducted in April 2019, June 2020, July 2021, and June 2022. Follow-up content was consistent with the baseline. The primary outcome of this study was the first diagnosis of CVD. To ensure the accuracy of the outcomes, we collected medical insurance and hospitalization records from 2016 to 2022. Information on the onset and timing of CVD was collected using the hospital’s social security information. If more than one CVD event occurred during the follow-up, only the first CVD event was included as an outcome event. For those who did not have a CVD event, the time of death or the last follow-up was used as the cut-off time for follow-up.

In this study, CVD was defined as ischemic heart disease (IHD) (International Classification of Diseases −10th revision, I20-I25) or stroke (I60-I64 and I69) during follow-up [21]. CVD events were recorded using self-reported questionnaires and hospitalization medical records. Self-reported patients had to provide proof of their clinical diagnosis.

### Statistical analysis

Continuous variables were described by mean ± standard deviation (SD), and categorical variables by frequency with percentage. Differences in characteristics across the cumBP groups were tested using the analysis of variance (ANOVA) or the Kruskal–Wallis test for continuous variables according to distribution and the Chi-square test for categorical variables. The Kaplan–Meier method was performed to compute the cumulative incidence of CVD, and the differences among groups were evaluated using the log-rank test.

Cox proportional hazard regression models were applied to calculate CVD’s hazard ratios (HRs) and 95% confidence intervals (CIs). The proportional hazard assumption was tested using Schoenfeld residuals. Three models were constructed: Model 1 was unadjusted; Model 2 was adjusted for age, gender, BMI, smoking, drinking, TG, TC, HDL-C, LDL-C, and FBG; and Model 3 was adjusted for the covariables of Model 2 plus baseline SBP and DBP. Standardized cumBP was included as an independent variable in the regression model to obtain the effect of each standard deviation increase in cumBP on the risk of CVD. *P*-values for trends were computed using the cumBP tertile as an ordinal variable. Restricted cubic spline (RCS) with four knots was used to capture the relationship between cumBP and CVD risk. When *P*_total_ < 0.05 and *P*_nonlinear_ > 0.05, indicating a linear relationship between the index and the risk of CVD. The multifactorial Cox regression analysis was repeated to examine the robustness of the results after excluding antihypertensive medication users, participants with a family history of CVD, and participants with diabetes, respectively.

After incorporating cumBP into the conventional CVD risk model (alternatively known as Model 3), we used the C-statistic, net reclassification index (NRI), and integrated discrimination improvement (IDI) to evaluate the incremental predictive value of cumBP. Furthermore, the receiver operating characteristic (ROC) curves were constructed to assess the predictive value of baseline BP and cumBP measurements for CVD risk.

All analyses were conducted using R software (version 4.2.2, 1.2, http://www.r-project.org/) and SPSS 26.0 (SPSS Inc., Chicago, IL, USA). Statistical significance was defined as a two-sided* P*-value < 0.05.

## Results

### Baseline characteristics of the participants

A total of 3,553 participants were included (mean age, 44.4 ± 12.5 years; 45.65% male). The baseline characteristics of the participants according to cumSBP tertiles are presented in Table 1. Compared with the lowest-tertile group, the participants in tertile 3 were more likely to be older, male, have a higher prevalence of diabetes, be more likely to take antihypertensive agents, have a higher BMI, TG, TC, LDL-C, FBG, SBP, DBP, MAP, PP, cumDBP, cumMAP, and cumPP levels, and lower HDL-C levels (*P* < 0.05, Table 1).

**Table 1:** Baseline characteristics of the participants by the tertile of cumSBP

Variables	Total (*N* = 3,553)	Tertile of cumSBP			*F*/χ^2^ value	*P*-Value
		Tertile 1 (*n* = 1,184)	Tertile 2 (*n* = 1,185)	Tertile 3 (*n* = 1,184)		
Age, years	44.4 ± 12.5	40.0 ± 10.3	42.3 ± 11.5 ^a^	50.9 ± 12.8 ^a, b^	294.93	< 0.001
Male, n (%)	1622(45.65)	463(39.10)	547(46.16) ^a^	612(51.69) ^a, b^	37.97	< 0.001
BMI, kg/m^2^	26.20 ± 4.66	24.98 ± 4.23	25.75 ± 4.52 ^a^	27.88 ± 4.72 ^a, b^	132.35	< 0.001
TG, mmol/L	1.71 ± 1.41	1.55 ± 1.37	1.68 ± 1.36	1.89 ± 1.46 ^a, b^	18.41	< 0.001
TC, mmol/L	4.70 ± 1.30	4.53 ± 1.28	4.68 ± 1.25 ^a^	4.88 ± 1.36 ^a, b^	22.12	< 0.001
HDL-C, mmol/L	1.45 ± 0.58	1.50 ± 0.65	1.45 ± 0.54	1.41 ± 0.53 ^a^	7.29	0.001
LDL-C, mmol/L	2.62 ± 0.94	2.54 ± 0.97	2.57 ± 0.91	2.73 ± 0.91 ^a, b^	14.38	< 0.001
FBG, mmol/L	4.95 ± 2.17	4.73 ± 2.16	4.90 ± 1.86	5.23 ± 2.42 ^a, b^	16.17	< 0.001
Smoking, n (%)	843(23.73)	250(21.11)	298(25.15)	295(24.92)	6.71	0.035
Drinking, n (%)	288(8.11)	85(7.18)	97(8.19)	106(8.95)	2.52	0.284
Diabetes, n (%)	204(5.74)	27(2.28)	54(4.56) ^a^	123(10.39) ^a, b^	76.52	< 0.001
Antihypertensive agent users, n (%)	191(5.38)	5(0.42)	20(1.69) ^a^	166(14.02) ^a, b^	262.73	< 0.001
Family history of CVD, n (%)	607(17.08)	195(16.47)	204(17.22)	208(17.57)	0.53	0.769
CVD events, n (%)	383(10.78)	64(5.41)	96(8.10) ^a^	223(18.83) ^a, b^	124.27	< 0.001
SBP, mmHg	128.16 ± 19.23	116.77 ± 12.67	124.45 ± 13.87 ^a^	143.25 ± 19.65 ^a, b^	892.11	< 0.001
DBP, mmHg	75.31 ± 11.50	70.64 ± 9.70	73.52 ± 9.73 ^a^	81.77 ± 11.88 ^a, b^	359.44	< 0.001
MAP, mmHg	92.93 ± 12.89	86.02 ± 9.40	90.50 ± 9.88 ^a^	102.27 ± 13.11 ^a, b^	699.30	< 0.001
PP, mmHg	52.85 ± 14.28	46.13 ± 11.21	50.93 ± 11.55 ^a^	61.48 ± 15.15 ^a, b^	448.37	< 0.001
CumSBP, mmHg·years	692.21 ± 107.90	584.93 ± 38.83	680.14 ± 23.95 ^a^	811.59 ± 83.35 ^a, b^	5097.03	< 0.001
CumDBP, mmHg·years	416.72 ± 62.05	362.72 ± 35.54	413.48 ± 33.83 ^a^	473.95 ± 54.26 ^a, b^	2058.19	< 0.001
CumMAP, mmHg·years	508.55 ± 74.16	436.79 ± 33.51	502.37 ± 26.46 ^a^	586.50 ± 58.44 ^a, b^	3819.93	< 0.001
CumPP, mmHg·years	275.50 ± 65.33	222.21 ± 31.62	266.65 ± 33.83 ^a^	337.63 ± 62.34 ^a, b^	1996.71	< 0.001

Data are presented as mean ± SD or n (%). The participants were stratified by tertiles of cumSBP: tertile 1, cumSBP ≤ 640.18 mmHg·years; tertile 2, 640.18 mmHg·years < cumSBP ≤ 723.92 mmHg·years; tertile 3, cumSBP > 723.92 mmHg·years

*Abbreviations*: *CumSBP* cumulative systolic blood pressure, *BMI* body mass index, *TG* triglycerides, *TC* total cholesterol, *HDL-C* high-density lipoprotein cholesterol, *LDL-C* low-density lipoprotein cholesterol, *FBG* fasting blood glucose, *CVD* Cardiovascular disease, *SBP* baseline systolic blood pressure, *DBP* baseline diastolic blood pressure, *MAP* baseline mean arterial pressure, *PP* baseline pulse pressure, *CumDBP* cumulative diastolic blood pressure, *CumMAP* cumulative mean arterial pressure, *CumPP* cumulative pulse pressure

^a^Compared with tertile 1, *P* < 0.05

^b^compared with tertile 2, *P* < 0.05

### Association between cumBP and the risk of CVD

**Fig. 1:**
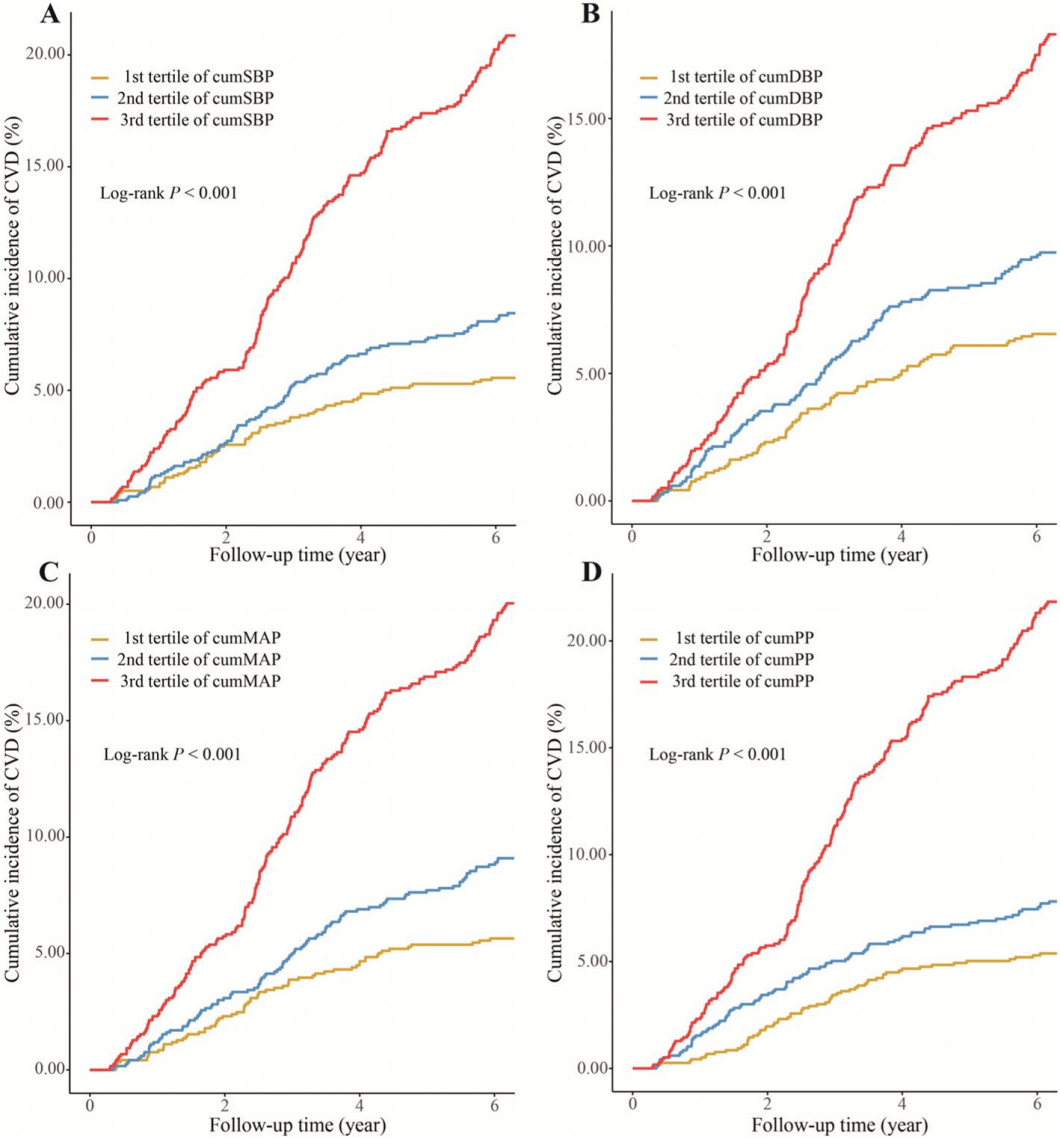
Cumulative incidence of CVD by tertile of cumBP. **A** CumSBP. **B** CumDBP. **C** CumMAP. **D** CumPP. Notes: The cumulative incidence of CVD by tertile of cumBP was calculated using the Kaplan–Meier method. The Log-rank test was used to calculate the *P*-value. The tertiles of cumSBP: tertile 1 (≤ 640.18), tertile 2 (640.18–723.92), tertile 3(> 723.92). The tertiles of cumDBP: tertile 1 (≤ 387.46), tertile 2 (387.46–436.54), tertile 3 (> 436.54). The tertiles of cumMAP: tertile 1 (≤ 473.59), tertile 2 (473.59–531.63), tertile 3 (> 531.63). The tertiles of cumPP: tertile 1 (≤ 242.61), tertile 2 (242.61–293.01), tertile 3 (> 293.01). CumBP is reported in mmHg·years. Abbreviations: CumBP, cumulative blood pressure; CumSBP, cumulative systolic blood pressure; CumDBP, cumulative diastolic blood pressure; CumMAP, cumulative mean arterial pressure; CumPP, cumulative pulse pressure. CVD, Cardiovascular disease

During a median follow-up of 6.29 years, 383(10.78%) incidents of CVD were identified, including 234 (6.59%) incident IHD and 155 (4.36%) incident strokes. The incidence of CVD increased substantially with the magnitude of cumBP (Supplementary Table 1). The probability of CVD events was significantly higher in participants with high cumBP than in those with low cumBP (*P* < 0.001, Fig. 1).

Table 2 shows the association of baseline BP and cumBP with CVD risk. Both baseline BP and cumBP were significantly associated with CVD risk in models 1 and 2, whereas a higher risk association was demonstrated for cumBP. The risk of CVD increased according to the magnitude of cumBP, and this trend remained significant even after adjusting for potential confounders in model 3 (*P* for trend < 0.05; Table 2). In model 3, cumSBP and cumPP showed the strongest association with CVD risk, and per 1-SD increase in them were associated with 31% and 28% higher risk of CVD, respectively [HR(95%CI): 1.310(1.153,1.489) and 1.284(1.132,1.457), Table 2).

**Table 2:** HR and 95%CI for the association between cumulative or baseline BP and CVD (per 1 standard deviation)

	Model 1		Model 2		Model 3	
	HR (95%CI)	*P*-value	HR (95%CI)	*P*-value	HR (95%CI)	*P*-value
SBP	1.630(1.506,1.765)	< 0.001	1.209(1.101,1.327)	< 0.001		
DBP	1.518(1.387,1.662)	< 0.001	1.205(1.096,1.325)	< 0.001		
MAP	1.626(1.495,1.768)	< 0.001	1.230(1.119,1.352)	< 0.001		
PP	1.487(1.362,1.623)	< 0.001	1.111(1.011,1.221)	0.028		
cumSBP	1.784(1.653,1.925)	< 0.001	1.317(1.202,1.443)	< 0.001	1.310(1.153,1.489)	< 0.001
*P* for trend	< 0.001		< 0.001		0.008	
cumDBP	1.616(1.483,1.760)	< 0.001	1.276(1.162,1.401)	< 0.001	1.219(1.074,1.384)	0.002
*P* for trend	< 0.001		< 0.001		0.044	
cumMAP	1.735(1.600,1.881)	< 0.001	1.313(1.196,1.441)	< 0.001	1.274(1.121,1.448)	< 0.001
*P* for trend	< 0.001		< 0.001		0.007	
cumPP	1.714(1.590,1.847)	< 0.001	1.245(1.139,1.362)	< 0.001	1.284(1.132,1.457)	< 0.001
*P* for trend	< 0.001		< 0.001		< 0.001	

Model 1: univariate Cox regression analysis; Model 2: adjusted for age, gender, body mass index, smoking, drinking, triglycerides, total cholesterol, high-density lipoprotein cholesterol, low-density lipoprotein cholesterol, and fasting blood glucose; Model 3: adjusted for model 2 covariates plus baseline systolic and diastolic blood pressure. The 1-SD increases for SBP, DBP, MAP, and PP correspond to 19.24 mmHg, 11.50 mmHg, 12.89 mmHg, and 14.28 mmHg, respectively. The 1-SD increases for cumSBP, cumDBP, cumMAP, and cumPP correspond to 107.90 mmHg·years, 62.05 mmHg·years, 74.16 mmHg·years, and 65.33 mmHg·years

*Abbreviations: HR* hazard ratio, *CI* confidence interval, *CumSBP* cumulative systolic blood pressure, *CumDBP* cumulative diastolic blood pressure, *CumMAP* cumulative mean arterial pressure, *CumPP* cumulative pulse pressure, *CVD* Cardiovascular disease

**Fig. 2:**
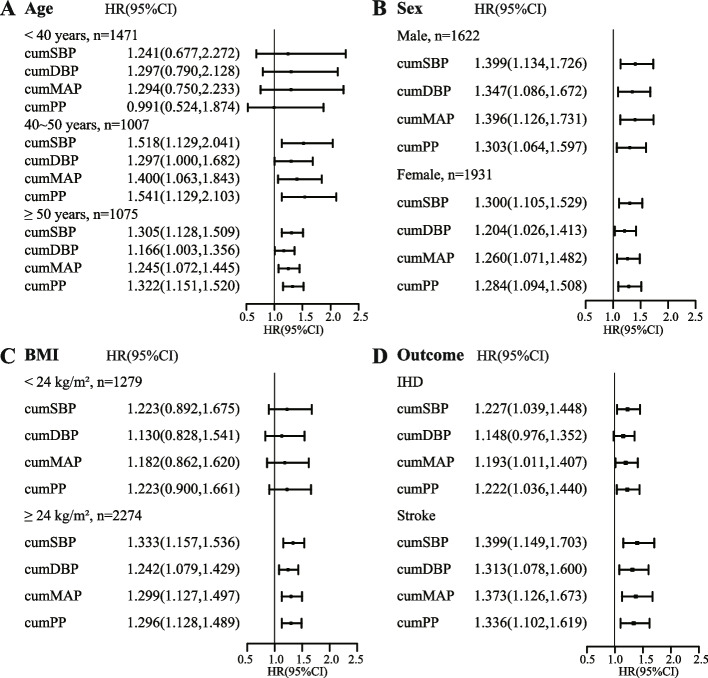
Association of cumBP with hazards of CVD subgroups analysis (per 1 standard deviation). The correction factors were the same as those in Table 2- Model 3. BMI < 24 kg/m^2^ was defined as normal weight, while BMI ≥ 24 kg/m^2^ was defined as overweight. Abbreviations: CumBP, cumulative blood pressure; CumSBP, cumulative systolic blood pressure; CumDBP, cumulative diastolic blood pressure; CumMAP, cumulative mean arterial pressure; CumPP, cumulative pulse pressure; CVD, Cardiovascular disease; IHD, ischemic heart disease

Subgroup analyses were performed after stratifying the study population by age, gender, and BMI. The results indicated that, among the four cumBP measures, cumSBP had the strongest association with CVD. The association between cumPP and CVD risk was higher in older individuals (age ≥ 40 years) and females, and cumMAP demonstrates a stronger association with CVD risk among males and individuals with a higher BMI. CumDBP was the weakest predictor of CVD risk among the four cumBP markers, but it was more important than cumPP in males (Fig. 2A, B, and C). Upon analyzing different CVD outcomes, it was observed that the four indicators of cumBP exposure exhibited stronger associations with stroke compared to IHD, with cumSBP exposure demonstrating the most potent correlation with stroke (Fig. 2D).

**Fig. 3:**
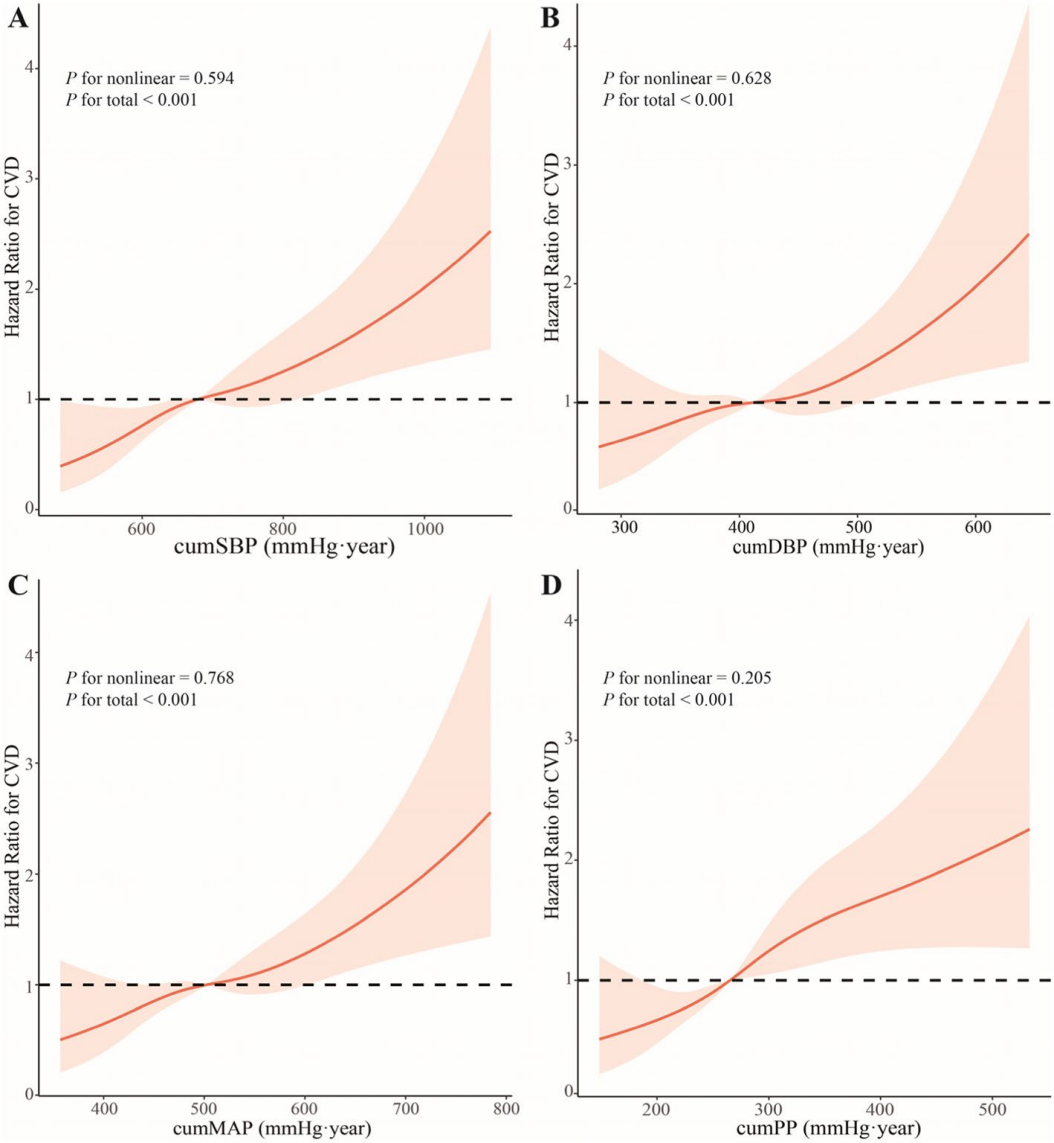
RCS plot of the cumBP and CVD risk. **A** CumSBP **B** CumDBP **C** CumMAP **D** CumPP. Notes: RCS analysis had four knots at the 5th, 35th, 65th, and 95th percentiles of the cumBP distribution. The model was adjusted for age, gender, body mass index, smoking, drinking, triglycerides, total cholesterol, high-density lipoprotein cholesterol, low-density lipoprotein cholesterol, fasting blood glucose, baseline systolic blood pressure, and baseline diastolic blood pressure. Abbreviations: RCS, restricted cubic spline; CumBP, cumulative blood pressure; CumSBP, cumulative systolic blood pressure; CumDBP, cumulative diastolic blood pressure; CumMAP, cumulative mean arterial pressure; CumPP, cumulative pulse pressure. CVD, Cardiovascular disease

Moreover, RCS analysis showed a linear relationship between cumSBP and CVD risk after adjusting for confounders (Fig. 3A). Similar results were found for cumDBP, cumMAP, and cumPP (Fig. 3B, C, D). In the sensitivity analysis, the association between cumBP and the risk of incident CVD was not materially changed after excluding antihypertensive agent users, participants with a family history of CVD, or diabetes, respectively (Supplementary Table 2).

### Incremental predictive value of the cumBP

Compared with the conventional CVD risk model, the conventional model combined cumSBP and cumMAP measurements obtained preferable C-statistics (0.808 for cumSBP, 0.807 for cumMAP). Additionally, incorporating cumSBP in the conventional model showed better risk reclassification power, with an NRI of 0.126(95%CI, 0.021–0.231; *P* = 0.019, Table 3). In terms of the IDI, all four cumBP measurements showed improved discriminatory capabilities, with cumSBP showing a superior effect in improving discrimination than the others (IDI: 0.009 for cumSBP, 0.005 for cumDBP, 0.007 for cumMAP, and 0.009 for cumPP).

**Table 3:** Incremental predictive value of the cumBP

	C statistics		NRI		IDI	
	Estimate(95%CI)	*P*-value	Estimate(95%CI)	*P*-value	Estimate(95%CI)	*P*-value
Conventional model	0.802(0.780,0.824)		Reference		Reference	
Conventional model + cumSBP	0.808(0.786,0.830)	0.026	0.126(0.021,0.231)	0.019	0.009(0.004,0.015)	0.001
Conventional model + cumDBP	0.805(0.784,0.827)	0.087	0.078(−0.028,0.183)	0.149	0.005(0.000,0.009)	0.029
Conventional model + cumMAP	0.807(0.785,0.829)	0.039	0.092(−0.014,0.197)	0.088	0.007(0.002,0.013)	0.005
Conventional model + cumPP	0.806(0.784,0.829)	0.067	0.095(−0.011,0.201)	0.080	0.009(0.003,0.014)	0.002

The conventional model was adjusted for age, gender, body mass index, smoking, drinking, triglycerides, total cholesterol, high-density lipoprotein cholesterol, low-density lipoprotein cholesterol, fasting blood glucose, baseline systolic blood pressure, and baseline diastolic blood pressure, alternatively known as Model 3. The NRI value refers specifically to continuous NRI

*Abbreviations: NRI* net reclassification index, *IDI* integrated discrimination improvement, *CumSBP* cumulative systolic blood pressure, *CumDBP* cumulative diastolic blood pressure, *CumMAP* cumulative mean arterial pressure, *CumPP* cumulative pulse pressure, *CVD* Cardiovascular disease, *CI* confidence interval

In addition, the areas under the ROC curves (AUC) were constructed to assess the predictive value of cumBP measurements for CVD risk. When directly comparing the predictive ability of baseline SBP and cumSBP for CVD risk, the AUCs were 0.652 and 0.692, respectively (95% CI: 0.621–0.682 and 0.662–0.721, *P* < 0.001, Supplementary Fig. 1A). Furthermore, upon incorporating cumSBP into the conventional model, the AUC increased from 0.802 to 0.808 (95%CI: 0.780–0.824 and 0.786–0.830, *P* = 0.026, Supplementary Fig. 1B).

## Discussion

In this cohort study of Chinese Uyghurs, we found that all four cumBP markers were significantly associated with CVD risk, especially among older individuals, those with a higher BMI and those who had previously experienced a stroke. The association between cumBP and CVD was stronger than that of BP at a single time point. Compared with the other three cumBP measures, cumSBP had the strongest association with CVD events and provided a superior incremental predictive value for CVD events.

Previous studies of the relationship between BP and CVD have focused on BP levels measured at a single time point, ignoring the disease risk associated with changes in BP over time. Long-term BP variability is a more critical determinant of cardiac damage than a single BP measurement [22]. Cumulative BP measures the level of fluctuations in BP over time, highlighting the vital influence of BP duration on the risk of developing CVD [23, 24]. Several studies have shown that cumSBP, cumDBP, cumMAP, and cumPP are all independent risk factors for CVD [5, 9, 10, 13], but few studies have compared which cumBP marker has better predictive efficacy.

The Coronary Artery Risk Development in Young Adults (CARDIA) study found that cumSBP and cumDBP were associated with incident CVDs [5], and the Lifetime Risk Pooling Project cohort study emphasized the importance of 10-year cumSBP as a risk factor for CVD over and above current SBP [13], which are consistent with our findings. Several studies have shown that cumBP provides incremental predictive and discriminatory value compared to a single time point BP assessment [5, 25, 26], and substituting long-term cumSBP for a single value can improve the ability of CVD risk prediction models [6]. In the present study, we found that adding cumBP to the conventional CVD risk prediction model had an incremental effect on the predictive value, with varying degrees of improvement in both NRI and IDI. CumSBP had stronger risk prediction performance than the other three cumBP markers, similar to the findings in the CARDIA study [5]. Because the effect of cumBP on CVD risk considers both the magnitude and cumulative duration of exposure to high BP, cumBP is a better predictor of the chronic effects of exposure on target organs. Using cumBP instead of single BP measurements may provide a more accurate prediction of the individual’s risk of developing CVD in this population.

MAP is determined by cardiac output and total peripheral arterial resistance, the pressure at which blood and oxygen flow steadily to peripheral tissues and organs [27]. One study claimed that MAP was a stronger predictor of CVD risk than SBP in men under 60 years of age [8], and another study conducted in an Iranian population showed that each standard deviation increase in MAP was associated with a 28% increase in the risk of CVD death [28]. Our study found that baseline MAP was associated with a 23% increased risk of CVD after adjustment for confounders in the COX regression model 2. The degree of association between MAP and CVD risk was similar to that of SBP. Recently, Wang et al. found that elevated cumulative MAP was an independent risk factor for ischemic stroke in hypertensive patients, especially in men over 60 years of age and in those with a higher BMI [10]. Additionally, results from a prospective cohort study of 53,813 subjects suggested that elevated cumulative MAP is an independent risk factor for adverse cardiovascular events [9]. Similar results were found in our research. We also found that cumMAP added predictive value to the traditional CVD risk prediction model, improving its C-statistic from 0.802 to 0.807, although only slightly improved (0.5%).

PP is critical in determining cardiovascular risk since PP is a marker of large artery stiffness [29]. A study reported that SBP or MAP showed the strongest relations to CVD, while relations of PP were less strong than those of SBP for CVD [30]. However, the Framingham Heart Study demonstrated that PP was the strongest predictor of coronary heart disease risk in individuals older than 50 [11]. This may be because PP and SBP become stronger predictors of coronary heart disease than DBP as DBP declines and PP rises steeply after age 50 to 60 years [12, 31]. A meta-analysis suggests that the risk of stroke and myocardial infarction in East Asian populations should be assessed primarily based on SBP, and MAP may also be a significant predictor, but PP is a less important predictor for CVD risk [23]. Cox regression analyses of baseline BP in this study showed similar results. The results of the CARDIA study showed that cumPP had lower magnitudes of association for clinical outcomes when compared to cumSBP and cumDBP [5]. Nevertheless, our findings suggest that cumPP was more strongly associated with CVD risk than cumDBP, with per 1-SD increase in them increasing CVD risk by 28% and 22%, respectively. Differences in the study populations may explain this. The former study was conducted in young white and African-American populations, whereas the present study was conducted in the Uyghurs in northwestern China, with higher mean PP values.

This study focused on the Uyghurs who lived in southern Xinjiang, China. Most of them are farmers and herders. They prefer salty milk tea, lamb products, and barbecued foods, and lack fresh vegetables and fruit intake. These characteristics contribute to the high prevalence of hypertension in this ethnic group, which results in a higher risk of CVD [15, 32]. Therefore, assessing and managing the long-term levels of variability in BP may play a crucial role in the prevention of CVD in this population.

The strength of this study is that it is the first to compare the predictive performance of four cumBP markers for CVD risk in the Uyghurs. Secondly, it takes into account the time effect in assessing CVD risk, which highlights the importance of long-term BP monitoring. Moreover, this study provides a new idea for constructing a CVD risk prediction model for this population. This study also has some limitations. First, the median follow-up time of this study was 6.29 years, which is relatively short and may not be sufficient to observe the full range of endpoint events. Second, there may be limitations in extrapolating the results of this study to other populations as it was conducted in the Uyghurs only. Finally, cumBP only marginally improved the predictive ability of CVD risk prediction models, and it remains essential to focus on the disease risk associated with a single BP measurement.

## Conclusion

This study found that four cumBP markers are all significantly associated with CVD risk. Cumulative BP has a higher predictive value than a single BP measurement. Compared with the other three cumBP measures, cumSBP has the strongest association with CVD events and provides a superior incremental predictive value for CVD events. CumBP may assist the risk stratification and individual prediction for future CVD events.

## Supplementary Information

Additional file 1: Supplementary Table 1. Cumulative incidence of CVD according to tertiles of cumBP. Supplementary Table 2. Sensitivity analysis for the association of cumBP with CVD (per 1 standard deviation). Supplementary Figure 1. (A) ROC analysis for baseline or cumulative SBP distinguishing CVD incidence. Supplementary Figure 1. (B) ROC analysis for including cumulative SBP to the conventional model predicting CVD incidence.

## Data Availability

The data used to support the findings of this study are available from the corresponding author upon reasonable request.

## Abbreviations

AUC: The Areas Under the ROC Curves
BMI: Body Mass Index
BP: Blood Pressure
CI: Confidence Interval
cumBP: Cumulative Blood Pressure
cumDBP: Cumulative Diastolic Blood Pressure
cumMAP: Cumulative Mean Arterial Pressure
cumPP: Cumulative Pulse Pressure
cumSBP: Cumulative Systolic Blood Pressure
CVD: Cardiovascular Disease
DBP: Diastolic Blood Pressure
FBG: Fasting Blood Glucose
HDL-C: High-density Lipoprotein Cholesterol
HR: Hazard Ratio
IDI: Integrated Discrimination Improvement
IHD: Ischemic Heart Disease
LDL-C: Low-density Lipoprotein Cholesterol
MAP: Mean Arterial Pressure
NRI: Net Reclassification Index
PP: Pulse Pressure
RCS: Restricted Cubic Spline
ROC: Receiver Operating Characteristic Curve
SBP: Systolic Blood Pressure
SD: Standard Deviation
TC: Total Cholesterol
TG: Triglyceride
