# Supplementary material for: Comparison of the cumulative exposure to four measures of blood pressure for predicting cardiovascular disease risk in the Chinese Uyghurs
Source: BMC Public Health. 2025 Mar 31;25:1214. doi: 10.1186/s12889-025-22069-9 (PMC11956206; doi:10.1186/s12889-025-22069-9)
Supplement: Supplementary file 1 — Additional file 1: Supplementary Table 1. Cumulative incidence of CVD according to tertiles of cumBP. Supplementary Table 2. Sensitivity analysis for the association of cumBP with CVD (per 1 standard deviation). Supplementary Figure 1. (A) ROC analysis for baseline or cumulative SBP distinguishing CVD incidence. Supplementary Figure 1. (B) ROC analysis for including cumulative SBP to the conventional model predicting CVD incidence. [file 12889_2025_22069_MOESM1_ESM.docx]

**Supplementary Material**

**Supplementary Table 1** Cumulative incidence of CVD according to tertiles of cumBP.

|  | Tertile 1 | | Tertile 2 | | Tertile 3 | | Log-rank  χ^2^ | *P*-value |
| --- | --- | --- | --- | --- | --- | --- | --- | --- |
|  | n (%) | Events/1000 person-years | n (%) | Events/1000 person-years | n (%) | Events/1000 person-years |  |  |
| cumSBP | 64(5.41) | 8.89 | 96(8.10) | 13.48 | 223(18.83) | 33.23 | 124.74 | <0.001 |
| cumDBP | 75(6.33) | 10.45 | 110(9.28) | 15.56 | 198(16.72) | 29.18 | 70.58 | <0.001 |
| cumMAP | 65(5.49) | 9.02 | 103(8.69) | 14.49 | 215(18.16) | 31.97 | 107.54 | <0.001 |
| cumPP | 62(5.23) | 8.57 | 89(7.51) | 12.49 | 232(19.61) | 34.74 | 147.85 | <0.001 |

**Notes:** The tertiles of cumSBP: tertile 1 (≤640.18), tertile 2 (640.18-723.92), tertile 3 (>723.92). The tertiles of cumDBP: tertile 1 (≤387.46), tertile 2 (387.46-436.54), tertile 3 (>436.54). The tertiles of cumMAP: tertile 1 (≤473.59), tertile 2 (473.59-531.63), tertile 3 (>531.63). The tertiles of cumPP: tertile 1 (≤242.61), tertile 2 (242.61-293.01), tertile 3 (>293.01). CumBP is reported in mmHg⋅years.

**Abbreviations:** CumBP, cumulative blood pressure; CumSBP, cumulative systolic blood pressure; CumDBP, cumulative diastolic blood pressure; cumMAP, cumulative mean arterial pressure; CumPP, cumulative pulse pressure. CVD, Cardiovascular disease.

**Supplementary Table 2** Sensitivity analysis for the association of cumBP with CVD (per 1 standard deviation).

|  | Excluded antihypertensive agent users | | Excluded participants with a family history of CVD | | Excluded diabetes | |
| --- | --- | --- | --- | --- | --- | --- |
|  | HR (95%CI) | *P*-value | HR (95%CI) | *P*-value | HR (95%CI) | *P*-value |
| Model 1 |  |  |  |  |  |  |
| SBP | 1.508(1.359,1.672) | <0.001 | 1.683(1.540,1.838) | <0.001 | 1.604(1.465,1.756) | <0.001 |
| DBP | 1.366(1.221,1.529) | <0.001 | 1.553(1.401,1.722) | <0.001 | 1.459(1.317,1.618) | <0.001 |
| MAP | 1.486(1.334,1.656) | <0.001 | 1.680(1.528,1.846) | <0.001 | 1.580(1.436,1.738) | <0.001 |
| PP | 1.369(1.230,1.524) | <0.001 | 1.540(1.399,1.696) | <0.001 | 1.483(1.343,1.637) | <0.001 |
| cumSBP | 1.703(1.542,1.881) | <0.001 | 1.840(1.686,2.007) | <0.001 | 1.780(1.631,1.943) | <0.001 |
| cumDBP | 1.466(1.316,1.633) | <0.001 | 1.649(1.495,1.819) | <0.001 | 1.604(1.457,1.767) | <0.001 |
| cumMAP | 1.611(1.452,1.788) | <0.001 | 1.788(1.629,1.961) | <0.001 | 1.722(1.571,1.887) | <0.001 |
| cumPP | 1.671(1.517,1.841) | <0.001 | 1.744(1.605,1.896) | <0.001 | 1.728(1.580,1.889) | <0.001 |
| Model 2 |  |  |  |  |  |  |
| SBP | 1.119(0.997,1.255) | 0.055 | 1.232(1.109,1.370) | <0.001 | 1.200(1.079,1.333) | 0.001 |
| DBP | 1.117(0.996,1.253) | 0.059 | 1.222(1.096,1.362) | <0.001 | 1.155(1.038,1.285) | 0.008 |
| MAP | 1.133(1.008,1.272) | 0.036 | 1.255(1.126,1.397) | <0.001 | 1.194(1.073,1.328) | 0.001 |
| PP | 1.059(0.947,1.183) | 0.314 | 1.129(1.016,1.254) | 0.024 | 1.136(1.021,1.264) | 0.019 |
| cumSBP | 1.248(1.114,1.398) | <0.001 | 1.326(1.195,1.472) | <0.001 | 1.338(1.205,1.486) | <0.001 |
| cumDBP | 1.181(1.055,1.323) | 0.004 | 1.285(1.155,1.430) | <0.001 | 1.284(1.156,1.425) | <0.001 |
| cumMAP | 1.223(1.092,1.371) | 0.001 | 1.325(1.191,1.474) | <0.001 | 1.325(1.193,1.472) | <0.001 |
| cumPP | 1.217(1.088,1.361) | 0.001 | 1.243(1.123,1.375) | <0.001 | 1.268(1.140,1.410) | <0.001 |
| Model 3 |  |  |  |  |  |  |
| cumSBP | 1.291(1.107,1.507) | 0.001 | 1.289(1.115,1.490) | 0.001 | 1.349(1.170,1.556) | <0.001 |
| cumDBP | 1.165(1.006,1.348) | 0.041 | 1.213(1.050,1.402) | 0.009 | 1.291(1.122,1.486) | <0.001 |
| cumMAP | 1.229(1.058,1.428) | 0.007 | 1.262(1.091,1.459) | 0.002 | 1.333(1.157,1.537) | <0.001 |
| cumPP | 1.315(1.125,1.537) | 0.001 | 1.254(1.088,1.445) | 0.002 | 1.282(1.110,1.481) | 0.001 |

**Notes:** The correction factors were the same as those in Table 2.

**Abbreviations:** CumBP, cumulative blood pressure; CumSBP, cumulative systolic blood pressure; CumDBP, cumulative diastolic blood pressure; cumMAP, cumulative mean arterial pressure; CumPP, cumulative pulse pressure. CVD, Cardiovascular disease; HR, hazard ratio; CI, confidence interval.


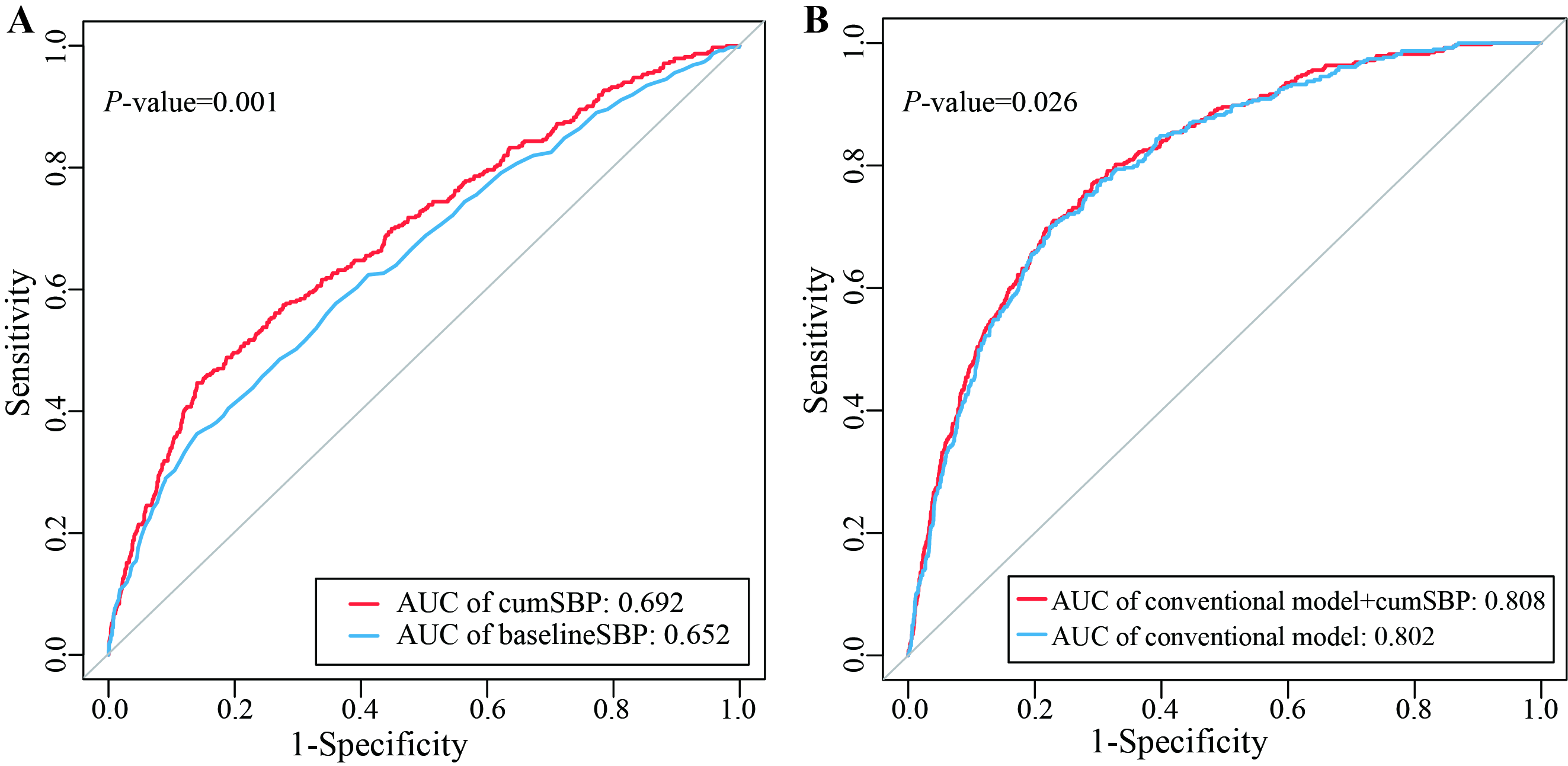


**Supplementary Figure 1** (A) ROC analysis for baseline or cumulative SBP distinguishing CVD incidence. (B) ROC analysis for including cumulative SBP to the conventional model predicting CVD incidence.

**Notes:** The conventional model was adjusted for age, gender, body mass index, smoking, drinking, triglycerides, total cholesterol, high-density lipoprotein cholesterol, low-density lipoprotein cholesterol, fasting blood glucose, baseline systolic blood pressure, and baseline diastolic blood pressure, alternatively known as Model 3.

**Abbreviations:** ROC, receiver operating characteristic; AUC, the area under the curve; CVD, cardiovascular disease; CumSBP, cumulative systolic blood pressure; SBP, baseline systolic blood pressure.
